# Technical Options and Airway Management in Carinal Resections

**DOI:** 10.3390/cancers18050844

**Published:** 2026-03-05

**Authors:** Peter Juhos, Miroslav Janík, Patrik Lauček, Jana Kudrnová, Róbert Baláž, Katarína Tarabová

**Affiliations:** 1 Department of Thoracic Surgery, University Hospital Bratislava, Slovak Medical University, Limbová 12, 833 03 Bratislava, Slovakiajana.szentivanyiova@gmail.com (J.K.); balaz.robert5@gmail.com (R.B.); 2 1st Department of Anaesthesiology and Intensive Care Medicine, University Hospital Bratislava, Faculty of Medicine, Comenius University, Špitálska 24, 813 72 Bratislava, Slovakia; tarabova@ru.unb.sk

**Keywords:** carinal resections, ECMO, crossfield ventilation, adenoid cystic carcinoma, lung cancer surgery

## Abstract

**Background**: Carinal resections remain a challenging and demanding surgical technique for both the patient and medical professionals. The most common indications are adenoid cystic carcinoma and bronchogenic carcinoma. There have been no randomized controlled trials because of the low incidence of pathologic processes suited to carinal resections and the difficulties associated with designing such studies. **Methods**: The known data are limited to a few single-institutional, retrospective studies over the last several decades. In this review article, we focus on the available data regarding surgical techniques and the types of ventilation that can help in the construction of the anastomosis—the most crucial part of the operation. Important issues regarding carinal resections are discussed in detail. **Results**: The available literature is reviewed in detail regarding indications, surgical techniques and approaches, types of ventilation, the rates of morbidity and mortality, and 5-year survival. The authors present their experience with two patients, where they utilized ECMO and crossfield ventilation. The role of minimally invasive surgery in carinal resections is also discussed. **Conclusions**: Carinal resections are complex surgical procedures, but acceptable mortality and morbidity rates can be achieved in carefully selected patients. Excellent cooperation between the surgeon and anesthesiologist is essential in the construction of the anastomosis. Various types of airway management, especially ECMO, help to reduce complication rates and facilitate secure airway reconstruction.

## Simple Summary

Carinal resections are rare but extremely complex procedures and represent a challenging topic, with a range of indications and types of reconstruction available via different approaches, from open surgery to minimally invasive techniques. Airway maintenance is crucial, and the choice of technique may help or hinder the construction of the anastomosis. It also has an impact on postoperative morbidity. Alternative techniques such as ECMO are being utilized more often in general thoracic surgery and are considered a feasible alternative in complex airway surgery. We aim to provide a comprehensive overview of this topic, covering key issues in carinal resections in the wider context of non-small cell lung cancer surgery.

## 1. Brief Summary of History of Carinal Resections

Historically, carinal resections were performed only in specialized high-volume centres, and there was a high rate of morbidity and mortality, including bronchopneumonias, arrhythmias, and anastomotic dehiscence or stenosis. The first attempts to resect the tracheal carina with a tracheobronchial anastomosis were performed in the 1940s in an experimental setting on dogs. Grindlay carried out a right carinal pneumonectomy with an end-to-end anastomosis of the distal trachea and the left mainstem bronchus [1]. The first clinical experience was presented by Abbott with four cases of carinal resection with extended pneumonectomy; he called this procedure a “tracheal sleeve pneumonectomy”. At a time when tumours invading the carina were considered inoperable, this was a major breakthrough [2]. Later in the 1950s, Crafoord reported the case of a 33-year-old patient with a tuberculosis stenosis where a right upper lobectomy was performed, and the right mainstem bronchus was resected with an anastomosis of the bronchus intermedius to the distal trachea [3]. In 1955, Bjork performed a resection of the left mainstem bronchus with an anastomosis of the secondary carina to the distal trachea [4]. In 1957, Barclay described a case of adenoid cystic carcinoma in a 24-year-old male patient involving the distal trachea, the carina, and both main bronchi. He performed a carinal resection with an end-to-end anastomosis of the distal trachea and right mainstem bronchus and an end-to-side anastomosis of the left mainstem bronchus to the bronchus intermedius. It was performed via a right posterolateral thoracotomy in a facedown position, ventilation was provided via a cuffed endotracheal tube, and then a cuffless tube was tightly inserted into the left main bronchus [5]. In 1963, Grillo presented a different reconstruction technique for use after the same type of resection for a “bronchial adenoma”. He performed an end-to-end anastomosis of the right mainstem bronchus to the distal trachea and an end-to-side anastomosis of the left mainstem bronchus to the trachea above the first suture line. The surgery was carried out through a combination of thoracotomy and sternotomy [6]. A cardiopulmonary bypass was first used by Woods et al. in 1961 for a carinal resection in a case of adenoid cystic carcinoma [7]. Echapasse described three cases of carinal resections in 1967 with a reconstruction technique opposite to that of Barclay and Grillo. In one case, he performed an end-to-end anastomosis of the distal trachea and the left mainstem bronchus and an end-to-side anastomosis of the bronchus intermedius to the left mainstem bronchus (reversed Barclay). In another case, he performed a similar reconstruction with an end-to-side anastomosis of the bronchus intermedius to the trachea just above the first suture line (reversed Grillo) [8].

## 2. Patient Selection

It is generally known that extended airway resections, especially carinal resections, with or without pneumonectomy, have high morbidity and mortality rates [9]. In order to keep perioperative morbidity and mortality as low as possible, careful patient selection is crucial. When a carinal resection with sleeve pneumonectomy is planned, patients are required to have sufficient pulmonary and general reserves during functional testing. Severe cardiac comorbidities may also impede extensive airway surgery. The same applies to carinal resections with lobar resections or lung-sparing techniques and neocarinal reconstruction. In the case of adenoid cystic carcinoma (ACC) extending to the carina or main bronchi, carinal resection is the primary treatment of choice. Although distant metastasis is less common, when it is present, extensive airway resection is contraindicated [10]. The aim in this surgery is to achieve a radical resection of the tumour and airway with an extent proportional to the technical possibilities of airway reconstruction. A microscopic R1 resection in the case of ACC is acceptable if adjuvant radiotherapy is feasible. If R2 resection is assumed during preoperative planning, it is considered a contraindication to surgery [11]. According to recent retrospective studies, patients with ACC after carinal resection have 5-year and 10-year survival rates of 81.5% and 60.2%, respectively [12]. The outcomes are poorer when microscopically positive margins are present (R1 resection). The 5-year survival in these patients is around 67%, especially when adjuvant radiotherapy is administered [11].

Preoperative staging, indication, and planning are more complicated with NSCLC (non-small cell lung cancer) involving the carina and the mainstem bronchi. As in less complicated cases of lung cancer, it is important to determine the N status. In patients who are considered for carinal resection, thorough preoperative staging is mandatory. N2 disease is generally considered a contraindication for surgery because of poorer outcomes and survival. We follow the guidelines provided by the European Society of Thoracic Surgeons on mediastinal staging [13]. A PET CT scan and EBUS or a videomediastinoscopy is carried out to determine the N status before extensive airway surgery. There is controversy regarding whether patients with single-station N2 disease could be candidates for surgery after induction therapy and successful downstaging [14]. Bronchoscopy is necessary to properly verify the extent of airway invasion. The margins of tumour extent should be verified histologically. Assessing the extent on imaging and bronchoscopy may be challenging, leading to it being underestimated. The most common indication for a right sleeve pneumonectomy is a tumour of the right lung involving the right upper lobar or intermedius bronchus that extends into the carina. Left carinal pneumonectomies are less common because of the length of the left mainstem bronchus. If the carina is invaded by the tumour, the mediastinal structures in the subaortic space and aortopulmonary window are also infiltrated. Unexpected scenarios may occur when positive margins are present in the frozen section after pneumonectomy, and carinal resections may be necessary [15].

## 3. Available Data

Studies on carinal resections are scarce and are mostly retrospective and single-institutional. One of the main reasons is the low incidence of pathological conditions suitable for this type of extensive surgery. Statistically valid randomization is difficult to achieve because of the low number of eligible patients, ethical concerns, and variability in surgical techniques, in combination with high morbidity. This is the reason why all recommendations and expertise are derived from retrospective and observational studies. We identified key published articles using OpenEvidence and Web of Science citation databases up to 2024 specifically for NSCLC and ACC separately. For NSCLC, studies where the surgical approach, type of ventilation, survival analysis, mortality and morbidity were stated were included. For ACC, we tried to identify all single-institutional series with at least 10 patients that included mainly carinal resections and stated the status of resection margins and survival analysis. The search was conducted using combinations of terms: airway surgery, carinal resections, crossfield ventilation, extracorporeal membrane oxygenation, non-small cell lung cancer, and adenoid cystic carcinoma. We performed a secondary analysis of the existing studies and publications and their bibliographies to be able to discover most of the papers regarding carinal resections.

To date, there are no meta-analyses or systemic reviews on this topic.

The majority of available studies on carinal resections for NSCLC are chronologically listed in Table 1, with a focus on the type of reconstruction, incisional approach, type of ventilation, mortality, morbidity, and survival.

Some of the most recent studies were analyzed in more detail.

In the most recent study, published in 2020, Sezen et al. evaluated 64 patients who underwent carinal resection for non-small cell lung cancer within an 11-year time span from 2005 to 2016 [16]. The data were prospectively collected, and long-term outcomes were analyzed. In total, 51 patients underwent right carinal pneumonectomy, and 13 patients underwent carinal sleeve lobectomy; 52 patients had squamous cell carcinoma, and 12 patients were diagnosed with adenocarcinoma. The preferred surgical approach was a right posterolateral thoracotomy in the fifth intercostal space. Crossfield ventilation was used to ventilate the contralateral lung after the transection of the airway. Of the patients, 5 underwent carinal resection because of unexpected intraoperative findings, and 59 underwent thorough lung cancer staging, including EBUS and videomediastinoscopy, for lymph node evaluation and careful preoperative planning. Complete resection was achieved in 56 patients (87.5%). Postoperative lymph node examination revealed N2 disease in seven patients (10.9%). Postoperative complications occurred in 31 patients (48.8%), mostly arrhythmias, ARDS (acute respiratory distress syndrome), hemorrhage, pneumonia, and bronchopleural fistula, and 7 patients died within 30 days after undergoing carinal pneumonectomy. The 2-, 5-, and 10-year survival rates were 64.9, 42.2, and 23.1%. The authors concluded that carinal resections for NSCLC are feasible, with acceptable morbidity and mortality, in carefully selected patients.

In 2019, Constantino et al. published a single-institutional report on carinal resections from 1997 to 2017 [17]. In total, 45 carinal resections were performed, with 21 neocarinal reconstructions, 14 right carinal pneumonectomies, 9 left carinal pneumonectomies, and 1 carinal sleeve lobectomy. In 15 patients (33%), the surgical approach was median sternotomy, whereas in 28 patients (62%), posterolateral thoracotomy was used. Ventilation was maintained via crossfield ventilation, and in four patients, V-A ECMO (extracorporeal membrane oxygenation) or cardiopulmonary bypass (CPB) was utilized. ECMO was also used postoperatively in two patients for respiratory failure and cardiogenic shock. In total, 53.3% of patients (24) had squamous cell carcinoma, adenocarcinoma, or carcinoid tumours, and 12 patients had adenoid cystic carcinoma. The overall 5-year survival for the entire cohort was 58%. The authors share their experience with ECMO and CPB as a planned operative strategy, particularly highlighting its benefits in tumours invading central vessels such as the aorta or superior vena cava, when standard crossfield ventilation is insufficient, and the advantages of utilizing this method in the postoperative period, when major complications occur.

Shin et al. analyzed 30 patients who received 17 right carinal pneumonectomies, 2 left carinal pneumonectomies, 2 neocarina reconstructions, and 9 carinal sleeve lobectomies over a period of 15 years (1996–2011) [18]. There were 22 cases of NSCLC and 8 cases of adenoid cystic carcinoma. Crossfield ventilation was employed in an intermittent fashion during the construction of the anastomosis. The endotracheal tube was left in place after surgery with minimal pressure, low tidal volume, and minimal oxygen for 1–2 days for airway secretion clearance. Thorough postoperative toileting of the airway was applied through bronchoscopy and chest physiotherapy. There was no death over the 30-day postoperative period. Pneumonia with ARDS occurred in three patients. Arrhythmias occurred in four patients, and three patients were treated for empyema after pneumonectomy or carinal sleeve lobectomy. These accounted for 36.6% morbidity. The 5-year overall survival was 66.3%. The relatively low complication rate and 0% mortality can be attributed to careful patient selection through preoperative diagnostic methods and the experience and cooperation of both the thoracic surgeon and the anesthesiologist.

Eichhorn and colleagues summarized their experience from Heidelberg between the years 2000 and 2011, including 64 patients with NSCLC, who received right carinal pneumonectomy in 61 cases and left carinal pneumonectomy in three cases [19]. Contralateral lung ventilation was maintained via high-frequency jet ventilation. In 83% of cases, complete R0 resection was achieved. All cases were discussed in the setting of a multidisciplinary tumour board. Induction therapy in the form of chemotherapy or chemoradiotherapy was administered to 8 patients, 18 patients received adjuvant chemotherapy, and 12 patients were treated with radiotherapy. In-hospital mortality was 3% (*n* = 2), and morbidity was 41%. Anastomotic leak, hemorrhage, and empyema were deemed to be major complications, with occurrence in five, two, and seven patients, respectively. Arrhythmias and respiratory failure were considered minor complications. The 5-year survival in the pN0 subgroup was 70%, but it was only 9% for pN2. In the definitive histopathology report, 26 patients had positive N2 nodes. All patients underwent invasive mediastinal staging via EBUS or videomediastinoscopy. Surgery was offered to biologically fit patients with curative but also palliative intent as part of a multimodality treatment approved by the tumour board and in close cooperation with the patient.

As listed in Table 1, the mortality rate tends to decrease over time, while morbidity is still high. There are many variables in between studies, such as ventilation maintenance, surgical approaches, and the heterogeneity of the cohorts. It is important to regard the interpretation of these data in the context of the time periods in which the treatment was carried out. The multimodality and individualized approach, with more options in terms of the neoadjuvant and adjuvant treatment of NSCLC patients, could have an impact on overall survival, especially in patients with positive lymph node involvement.

Concerning adenoid cystic carcinoma, in a recent study in France, Estephan et al. shared their single-institutional experience with 59 patients over a time span of 49 years [12]. Out of these patients, 38 received complex surgery, with 9 carinal resections and 12 carinal pneumonectomies. The other surgeries included total tracheal replacement and tracheal resection with laryngectomy. The surgical approach comprised a posterolateral thoracotomy or sternotomy. In the complex surgery group, R0 resection with clear margins was achieved in 8 patients (21%), R1 resection in 28 patients (73.7%), and R2 resection in 2 patients (5.3%). Major complications were present in 13 patients (34.2%) in the complex surgery group. There was no 30-day mortality recorded among the patients who underwent carinal resection. The 5-year overall survival in the whole cohort was 81.5%. The overall survival was not significantly different between the standard and complex surgery groups. Patients with T4 and R1 and R2 resections had poorer survival in comparison with the R0 group. Patients with R1 resection who received adjuvant radiotherapy had significantly better 5- and 10-year survival rates than those without radiotherapy. The authors found that a dedicated and experienced team specialized in tracheal surgery could help in achieving optimal outcomes. Moreover, preoperative planning and imaging techniques, as well as neo- and adjuvant treatments, have changed over the last 50 years. The authors concluded that R0 resection may involve excessive risks to the patient and should not be sought at all costs, particularly as radiotherapy after R1 resection is effective.

A list of all the relevant studies concerning ACC and carinal resections with 5- and 10-year survival and recurrence rates is provided in Table 2.

## 4. Complications of Carinal Resections and How to Avoid Them

Carinal resections, whether lung-sparing or with a pneumonectomy or lobectomy, pose the challenge of high perioperative mortality and morbidity. These procedures are technically demanding not only for the thoracic surgeon but also for the anesthesiologist and intensivist. Precise patient selection is necessary to lower the complication rate. To be able to properly select suitable candidates for surgery, numerous diagnostic procedures must be performed.

According to the literature, the overall complication rate for carinal resections varies between 35 and 58%, with perioperative mortality rates around 10% [17]. The most common complications include pneumonia, atrial arrhythmias, anastomotic complications (such as dehiscence or stenosis), acute respiratory distress syndrome, empyema, and hemorrhage. The most serious are anastomotic complications with high mortality rates. Anastomotic complications occur at a rate of approximately 14%, and 50% are fatal [30]. In the study by Sezen, bronchopleural fistula/anastomotic leak was present in 9.8% in the carinal pneumonectomy group and in 7.7% of cases in the carinal sleeve lobectomy group. This complication proved fatal in the pneumonectomy group in comparison with the lobectomy group. The authors believe that lung parenchyma preservation prevented mortality in this group [16]. Factors that affect the development of bronchopleural fistula/anastomotic dehiscence include the disruption of carinal and bronchial blood supply due to excessive dissection, anastomotic tension due to the large segment of the airway resected, and inaccurate suturing techniques and technical errors. Anastomotic complications range from excessive granulation, necrosis, bronchial mucosal sloughing, and microfistula to the more complex and life-threatening complete dehiscence of the anastomosis, characterized by almost 100% mortality [30].

Numerous studies have demonstrated poor anastomotic healing after neoadjuvant therapy [17]. Tumour-positive resection margins have a negative influence on the patency of the anastomosis. However, in the previously mentioned study by Sezen, there was no statistically significant correlation between neoadjuvant therapy and morbidity and mortality.

Postoperative complications seem to be associated with lower DLCO values, although previous lung resection is not associated with increased risk. ARDS and anastomotic complications represent the main causes of death due to respiratory failure [31]. Acute respiratory failure usually develops in the first 72 h postoperatively. The cause is multifactorial; ventilator-induced barotrauma and fluid overload during surgery are well-known risk factors.

Advances in anesthetic techniques and multiple options for ventilation, as well as improved intensive care, have improved outcomes, but patient selection remains the cornerstone of minimizing the occurrence of complications.

Preoperative bronchoscopy is performed to identify tumour extent and histological type. Chest computed tomography (CT) scans with contrast are also routinely performed. Pulmonary function testing and lung ventilation/perfusion scans should be used to evaluate and predict postoperative pulmonary functions. Electrocardiography, echocardiography, and an exercise test should be performed to assess cardiac function. Magnetic resonance imaging of the brain and PET CT scan are used for the detection of distant metastasis.

After a suitable candidate for surgical treatment is selected, there are several intra- and perioperative steps that help in avoiding complications.

It is important for the anesthesiologist to be knowledgeable in the steps of the surgical procedure and be well versed in optional ventilatory techniques, including HFJV (high-frequency jet ventilation) and one-lung ventilation. Close cooperation between the anesthesiologist and surgeon allows for the intermittent ventilation of the lung utilizing crossfield ventilation, HFJV, apneic preoxygenation techniques, or ECMO management. Frozen section analysis of resected airway margins by a competent pathologist must be available.

The procedure must be performed by an experienced thoracic surgeon. To minimize the risk of anastomotic dehiscence or stenosis, anastomotic tension should be avoided, tracheobronchial blood supply must be carefully preserved, the use of release maneuvers is encouraged, and airway anastomosis should be reinforced with vascularized tissue flaps. The length of the resected trachea and mainstem bronchus should not exceed 4 cm when carinal pneumonectomy is performed.

Mulliken and Grillo showed that with 15° to 35° of neck flexion, a 4.5 cm length of the trachea can be resected with a tension-free primary anastomosis [32]. Two stiches are placed from the submental area to the anterior chest.

The avascular pretracheal plane is bluntly dissected, taking special care to avoid injury to the lateral blood supply of the trachea. This could be done through a transsternal approach, or when the carina is resected through a right thoracotomy, a prior mediastinoscopy is performed. Laryngeal and suprahyoid release maneuvers are not recommended when the distal trachea or carina are resected. Right hilar and pericardial release are more beneficial in lower tracheal surgery. The pulmonary ligament is divided, and a U-shape incision is made around the inferior pulmonary vein posterior to the phrenic nerve. Neck flexion together with pretracheal blunt dissection and hilar and pericardial release may allow us to resect nearly 50% of the trachea [33].

During the procedure, barotrauma to the airway should be avoided, as well as fluid overload, hypoperfusion, and prolonged mechanical ventilation. These factors increase the risk of development of ARDS.

Early extubation, airway clearance with bronchoscopy, chest physiotherapy, and monitoring for arrhythmias are key elements in avoiding pneumonia and respiratory failure in the early postoperative period.

The timely recognition and management of bronchopleural fistula and anastomotic dehiscence is crucial in order to prevent mortality. Some authors have described their experience with stenting or hyperbaric oxygen therapy when there is necrosis at the anastomotic site.

Technical innovations and the advent of minimally invasive procedures such as VATS (Video-assisted Thoracic Surgery) and RATS (Robotic-assisted Thoracic Surgery) may reduce the anastomotic and overall complication rates.

## 5. Anastomosis and Suturing Techniques

The aim of each anastomotic technique is to achieve a tension-free anastomosis of the trachea and the bronchi with clear surgical margins when possible. The anastomosis must be checked for any air leakage at the end of the procedure. It is important to preserve the blood supply of the trachea in order to prevent catastrophic complications such as dehiscence. The maximum extent of resection between the trachea and the opposite bronchus should be no longer than 4 cm to achieve a tension-free anastomosis.

Interrupted absorbable sutures are placed circumferentially to allow for the adjustment of different diameters of the connecting airways. This technique is considered the gold standard for open carinal resections.

Multiple absorbable running sutures can be applied to construct the anastomosis. Some surgeons consider this technique to be less technically demanding and faster than interrupted suturing [34].

Neocarina reconstruction is a technique used when only a limited part of the carina is involved, with no spread to the main bronchi. The medial walls of the bronchi are sutured together, creating a neocarina that is connected to the distal trachea.

A bronchial flap method has also been described, with omental flap reinforcement [35].

We prefer the use of synthetic monofilament absorbable sutures such as maxon, biosyn, or prolene.

## 6. Utilization of ECMO in Different Surgical Approaches and Reconstruction Types

Different tumour localizations necessitate resection to different extents, which can be achieved via various approaches. The crucial part is the airway reconstruction, which requires meticulous surgical skill and a competent and experienced anesthesiologist to maintain ventilation. This can be achieved through various techniques described in this article. ECMO greatly helps during this phase, since there is no physical tube in the operating field and airways.

Left carinal pneumonectomy is rarely indicated, as is shown in Table 1. The reason for this is the anatomical length of the left main bronchus. If there is tumour spread into the carina, this usually suggests the infiltration of the mediastinal and hilar structures, as well as the subaortic space, suggesting inoperability [15]. If only a small part (1 cm) of the carina and contralateral bronchus needs to be resected, then left posterolateral thoracotomy is the standard approach. If the resection of longer segments of the distal trachea or contralateral bronchus is necessary, then a complete median sternotomy is the recommended approach. The aortic arch needs to be mobilized and the ductus arteriosus transected. The left hilar vessels are divided first, followed by left mainstem bronchus and carinal resection. At this point in the operation, the tube is placed in the right main bronchus, usually via crossfield ventilation or HFJV. It is strongly recommended that ECMO support be used in this phase to facilitate superior conditions for the construction of the anastomosis between the distal trachea and the right mainstem bronchus [36].

Right carinal pneumonectomy is more frequent, and the most common indication is a tumour originating in the right upper lobe bronchus and extending to the distal trachea. The standard approach for this procedure is right posterolateral thoracotomy. At most, four centimetres of the distal trachea should be resected to achieve a tension-free anastomosis and to avoid dehiscence and life-threatening fistulation. Fat pad reinforcement of the anastomosis is also recommended. The left main bronchus is transected first, and either crossfield ventilation or ECMO can be applied for better conditions.

Neocarina reconstruction is usually indicated in the case of adenoid cystic carcinoma, as is demonstrated in our case. This technique is applied when small carinal tumours without main bronchial infiltration are present. The standard incision involves a median sternotomy and transpericardial approach. The reconstruction itself is performed by suturing the medial parts of the main bronchi together and then connecting them to the distal trachea [9]. V-V or V-A ECMO can greatly help during the construction of the anastomosis.

Regarding carinal resections without lung resection, in the technique by Barclay, Grillo, and Eschapasse, the distal trachea is first connected via an end-to-end anastomosis to the right or left mainstem bronchus, and then a second end-to-side anastomosis is constructed to either the trachea, the bronchus intermedius, or the left mainstem bronchus. These procedures are carried out using a median sternotomy and transpericardial approach. The construction of the second anastomosis is even more complicated, with crossfield ventilation. V-V ECMO is a helpful alternative in these cases [37].

## 7. Minimally Invasive Carinal Resection and Reconstruction

Open approaches such as sternotomy and thoracotomy remain the gold standard in carinal resection cases. New minimally invasive methods are emerging, such as VATS and RATS carinal resections. Currently, there are no large studies evaluating these techniques, but there is evidence in the form of case reports with successful results. These papers suggest that a minimally invasive technique is feasible, albeit demanding for the surgeon. Some authors even claim that there is a lower risk of postoperative morbidity [38]. One of the few studies, published in 2020 by Sekhniaidze and Gonzalez-Rivas, describes the authors’ technique for carinal resection using the uniportal VATS approach on 16 patients with NSCLC [39]. In total, 11 patients (69%) underwent right carinal pneumonectomy, 4 (25%) underwent carinal sleeve right upper lobectomy, and 1 underwent a lung-sparing carinal resection (6%); 7 patients received neoadjuvant chemotherapy, and 10 underwent adjuvant chemotherapy. Postoperative complications occurred in four patients. One patient was treated for pneumonia, and there was one case of chylothorax that needed thoracic duct clipping, one case of hemothorax, and one case of anastomotic fistula that needed an urgent thoracotomy with the suturing of the anastomosis and diaphragmoplasty. The authors used a vascularized diaphragmatic flap for anastomotic covering in three additional patients where the risk of anastomotic leak was higher. There was no mortality recorded. The anastomosis was constructed in an end-to-end fashion using an absorbable 3/0 prolene running suture or a double-barrel technique known from sleeve lobectomies. In the case of carinal sleeve lobectomies, the reconstruction of the secondary carina was technically more difficult. Ventilation was maintained using a double-lumen endobronchial tube, and after the transection of the airway, high-frequency jet ventilation was applied.

The authors attribute the low complication rate to careful patient selection and the general benefits of the minimally invasive approach. They also highlight the direct line of sight and instrumentation through the uniportal incision, which is identical to that in open surgery.

Besides the above-mentioned work of Sekhniaidze and Gonzalez-Rivas, there is only one series of VATS carinal resection and reconstruction with 12 patients from three centres in China. These authors used a multiportal VATS approach that allowed them to use crossfield ventilation. In this small series, no in-hospital mortality or major morbidity occurred. They compared their results with the thoracotomy approach, where postoperative mortality ranged from 2.4 to 12.7% [38].

RATS carinal resections and reconstructions are only seen in the form of case reports, and there are no larger series reported.

While in general, VATS techniques show significant benefit over open surgery in regard to postoperative or chronic pain and quality of life, which is backed by randomized controlled trials, evidence of superiority in regard to specific peri- or postoperative morbidity and mortality in the case of carinal resections is absent. Since only two series and case reports are recorded in the literature, where patients are subjected to selection bias, the efficacy of minimally invasive methods should be considered with caution until further, more comprehensive data are available.

## 8. Different Types of Ventilation

The success of carinal resections and airway reconstruction surgery depends on flawless anesthesia and airway management, which provide excellent conditions for anastomosis construction and lower the risk of peri- and postoperative complications. Crossfield ventilation is the gold standard and the most frequently used method, especially in open approaches such as a thoracotomy or sternotomy. A reinforced endotracheal tube is placed into the contralateral bronchus after the transection of the airway and is connected to the ventilator through a sterile circuit across the operating field [40]. The main setback is the need for the repeated withdrawal and reinsertion of the tube into the bronchus to facilitate apneic preoxygenation ventilation. This interferes with the construction of the posterior wall of the anastomosis, obstructs the surgical view, and may cause damage to the airway. The use of crossfield ventilation is even more difficult in minimally invasive approaches. There is a lack of consensus in these cases. An alternative to crossfield ventilation is high-frequency jet ventilation (HFJV). This is an established ventilation method in open or minimally invasive airway surgery and interventional bronchoscopic procedures. Its advantages include the minimal obstruction of the operating field while maintaining oxygenation with low airway pressure and a low tidal volume. The baseline parameters are as follows: FiO2 = 90%; frequency = 100–120 per minute; pressure range = 0.9–1.2 bar. It can be used as a rescue technique when crossfield ventilation fails. It may also be used in non-intubated procedures in selected patients. Since indications for carinal resections are scarce, there are few studies comparing airway management. The only retrospective study comparing crossfield ventilation and HFJV is from 2022, by Qiu and colleagues from Shanghai Chest Hospital. They conducted a retrospective analysis on 22 patients who underwent carinal resections between 2018 and 2021 and were divided into two groups according to ventilation management. There were no statistically significant differences between the two groups regarding the severity and duration of intraoperative hypoxemia. The cumulative time of SpO_2_ < 90% was 16.67 min in the crossfield ventilation group and 19.32 min in the HFJV group. Severe intraoperative hypoxemia (SpO_2_ < 80%) did not occur under either technique. Since EtCO_2_ monitoring is not reliable when using either crossfield ventilation or HFJV, the authors intermittently checked blood gas. They also expressed their belief that with the advent of minimally invasive carinal resections using VATS/RATS techniques, HFJV may become the new standard for airway management.

Using ECMO as a means of ventilatory support during complex tracheobronchial resections has numerous advantages, and as described in this paper, it represents an ideal alternative to mechanical ventilation techniques. V-V ECMO is the most common method in thoracic surgery when hemodynamic support is not needed. The aim of V-V ECMO is to achieve sufficient ventilation and CO_2_ removal. Lang et al. published their experience with V-A ECMO in a cohort of 10 patients with NSCLC with no mortality and a complete resection rate with negative margins of 89%. The previously mentioned methods may cause blood aspiration to the contralateral lungs while intermittently removing the armed tube to the contralateral bronchus, as well as ischemia of the proximal part of the transected bronchus through balloon overinflation. Hypercapnia is often present, as well as atelectasis, leading to a higher risk of postoperative pneumonia or ARDS. With the absence of ventilation tubes in the surgical field, the construction of the anastomosis is more efficient and reduces the risk of dehiscence with fatal consequences due to technical flaws. The mean duration of ECMO assistance was 38 min in the series reported by Spaggiari [41]. The risk of tumour cell metastasis is minimal since ECMO is only applied after the vessels are ligated and the lung parenchyma is removed. Anticoagulation is limited, and coagulation parameters quickly return to normal, so postoperative hemorrhage is rare.

The above-mentioned series favour the application of ECMO; however there is no consensus on the absolute indications for using ECMO in thoracic surgery and especially in carinal resections [42,43]. The technical advantages of different types of carinal resections from the surgeon’s perspective were described in this paper, but this method should still be considered with caution regarding potential complications. Patients who may benefit from ECMO support are those who lack pulmonary reserve or do not tolerate one lung ventilation, e.g., obese patients or patients with chronic obstructive pulmonary disease or cardiac comorbidities, as well as patients undergoing surgery that will involve the retraction of the heart, mediastinum, and large vessels [44,45].

V-V ECMO is predominantly deployed to provide respiratory support, while V-A ECMO should be utilized if there is hemodynamic instability and circulatory support is needed. The surgical approach may also determine the more suitable method. V-A ECMO is better suited for carinal resections via the transsternal approach where the mobilization of large vessels and the heart is necessary, while V-V ECMO is preferable when carinal resection through right thoracotomy is performed [46].

Potential major complications involve bleeding, vascular injury, recirculation syndrome, and cannula-related problems when the blood flow through the cannula is obstructed by the collapsed vessel wall or hypovolemia. Weaning the patient from ECMO after the surgery is completed should prevent the need for reoperations for bleeding. With V-V ECMO, lower heparin doses are required, and some centres are considering excluding heparin in the setting of intraoperative V-V ECMO utilization [47].

**Case 1**

**Figure 1:**
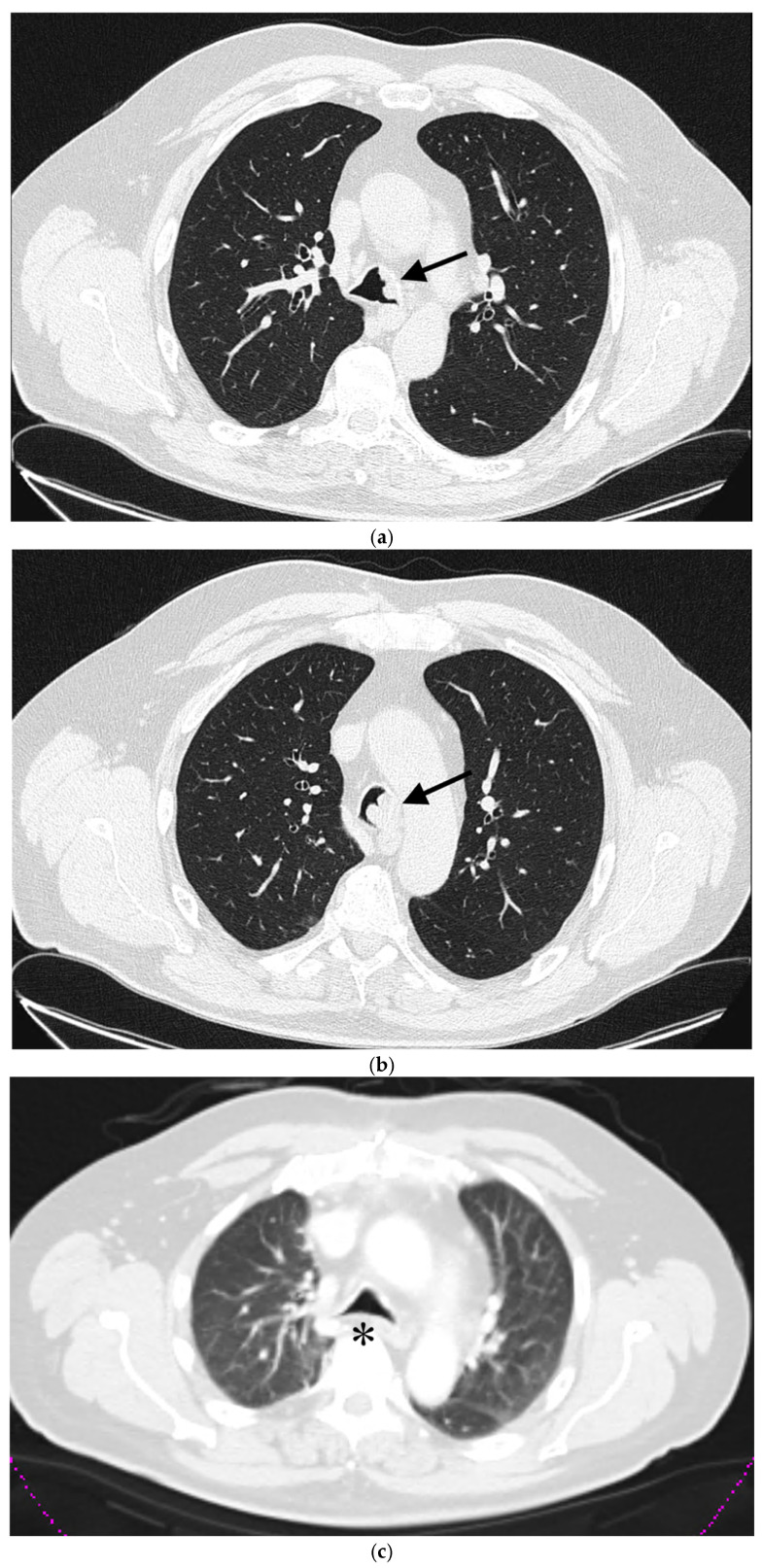
(**a**) A CT scan of a tumorous mass in the distal trachea, (**b**) tumorous mass in the carina, (**c**) postsurgery CT. arrow—indicating the localization of the tumour, * reconstructed tracheal carina.

**Figure 2:**
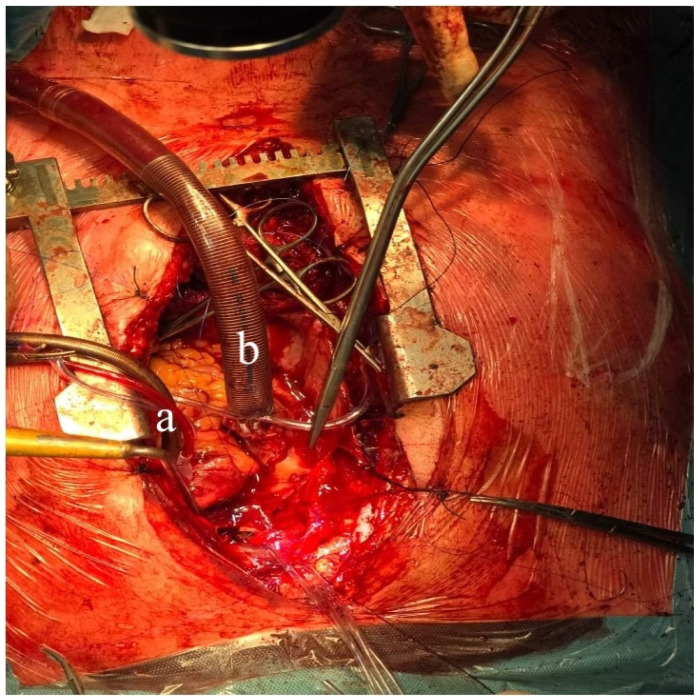
a—arterial cannula in the aorta; b—venous cannula in the right atrium.

**Figure 3:**
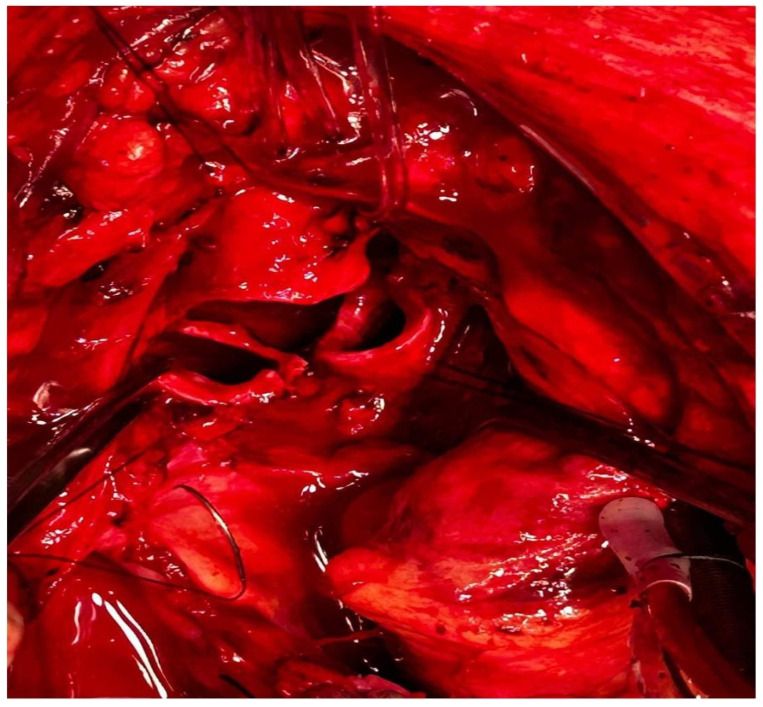
Transected distal trachea, right and left mainstem bronchi with traction sutures, retracted aorta, and left brachiocephalic vein.

**Figure 4:**
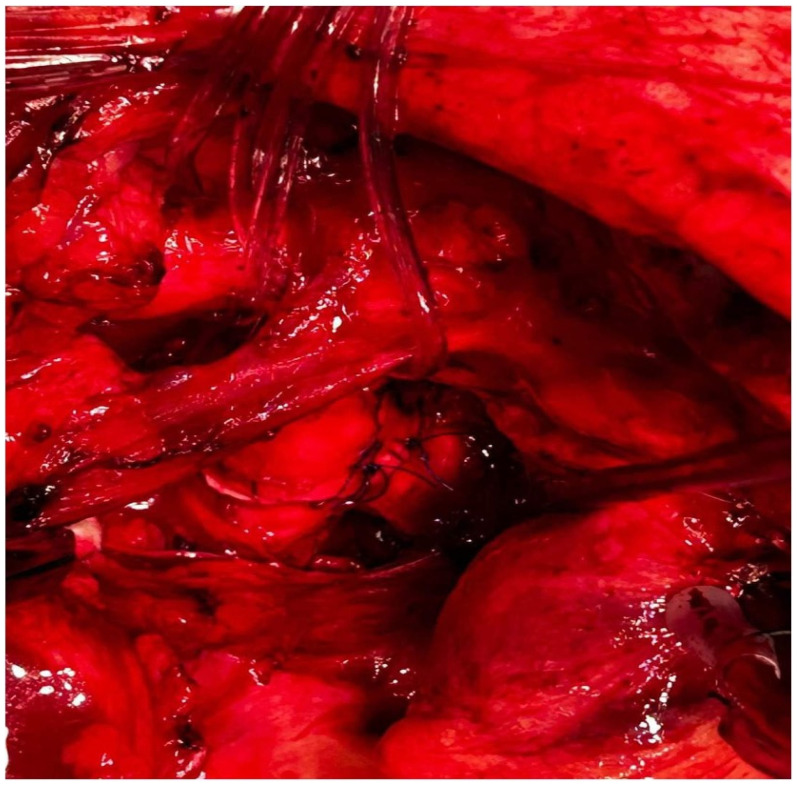
Complete anastomosis—neocarina.

A 60-year-old patient with COPD (chronic obstructive pulmonary disease) and hypertension was admitted to a secondary hospital for worsening dyspnea and stridorous breathing. A CT scan revealed a mass in the distal trachea and its bifurcation obturating 70% of the tracheal lumen (Figure 1a,b). The patient was urgently referred to a tertiary centre, where the bronchoscopic recanalization of the airway was performed and histology acquired. The pathology report disclosed the presence of an adenoid cystic carcinoma. Cardiopulmonary functional testing was carried out in an elective setting. VC was 103%, FEV1 70%, and DLCO 93%. The ejection fraction of the left ventricle on echocardiography was 55%. Several PET CT scans were performed, and all the preoperative procedures were analyzed by a multidisciplinary tumour board. A median sternotomy with a transpericardial approach was planned for resection and neocarinal reconstruction. V-V ECMO was decided on for maintaining ventilation. Due to technical difficulties in femoral and jugular vein cannulation, V-A ECMO was utilized directly through the auricle of the right atrium and the aorta (Figure 2). The flow rate was gradually raised to 5.2 L/min, FiO2 0.6, rpm 3240. A tumour infiltrating approximately 2.5 cm of the left wall of the distal trachea and left mainstem bronchus was resected in macroscopically negative margins (Figure 3). Even after release maneuvers, this was the limit of resection for a safe, tension-free anastomosis construction. First, the medial walls of the main bronchi were sutured together and connected to the distal trachea with a 3/0 prolene running suture (Figure 4). After 35 min, when airway reconstruction was completed, standard ventilation via an endotracheal tube was resumed. The patency of the anastomosis was examined via bronchoscopy. Subcarinal lymph nodes were dissected and removed. Traction sutures of the neck and chest were applied for neck flexion to lower the tension to the anastomosis. The patient was extubated in the operating theatre and observed in the ICU for 72 h without complications. On the seventh postoperative day, a follow-up bronchoscopy showed no sign of dehiscence or anastomotic fistula. The traction sutures were removed and the patient discharged. The definitive pathological report revealed positive resection margins without nodal involvement, and the patient received adjuvant radiotherapy. Almost 2 years after surgery, the patient is without locoregional or distant relapse. The patency of the neocarina was repeatedly verified through bronchoscopy and imaging techniques (PET CT) (Figure 1c).

**Case 2**

**Figure 5:**
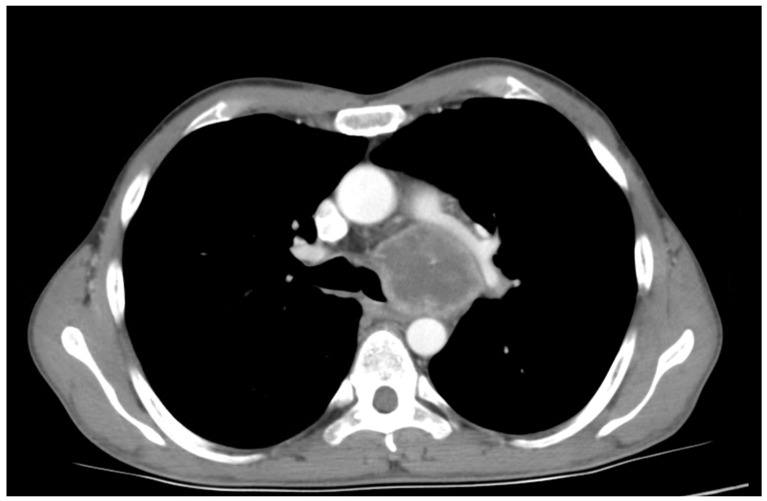
Initial CT scan showing a tumorous mass infiltrating the distal trachea and left mainstem bronchus.

**Figure 6:**
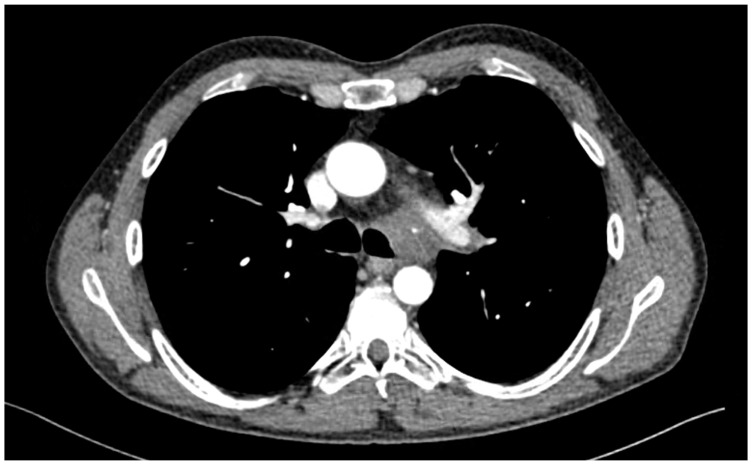
CT scan after induction therapy.

A 41-year-old male patient was presented with a fever, coughing, and worsening dyspnea. A chest X-ray revealed a mass in the left hilar and mediastinal region. The patient was admitted to the hospital, where a CT scan of the chest showed a tumorous mass encompassing and infiltrating the left mainstem bronchus and invading the mediastinum (Figure 5). Bronchoscopy disclosed stenosis and a tumorous infiltration of the left mainstem bronchus. The pathology report showed the presence of lung adenocarcinoma; for further genetic evaluation, a cryobiopsy was performed. The patient received six cycles of induction chemotherapy consisting of cisplatin and vinorelbin. After three cycles, concomitant radiotherapy was administered with a dose of 64 Gy. A reduction in tumour size and infiltration was noted on a follow-up CT scan and bronchoscopy (Figure 6). Radical resection was indicated by a multidisciplinary tumour board, with an extent up to left carinal pneumonectomy. Functional lung testing showed a VC of 88%, FEV1 of 87%, and DLCO of 52%. The surgery was carried out through a left posterolateral thoracotomy. The pulmonary veins and pulmonary artery were divided intrapericardially. The tumour was meticulously dissected from the subaortic space and the aortic arch. During this phase, a tear in the aortic arch was sutured without substantial blood loss. The left mainstem bronchus and lateral wall of the distal trachea were resected in a wedge fashion. The right mainstem bronchus was intubated with a reinforced orotracheal tube, size 6, using the crossfield ventilation method. A running suture with prolene 3/0 was used to anastomose part of the distal trachea and the right mainstem bronchus. Orotracheal ventilation was resumed, and the patency of the anastomosis was checked under water and with bronchoscopy. The patient was extubated in the operating theatre and observed in the ICU for 48 h without complications. The patient was discharged on the 10th postoperative day, after antibiotic treatment, and follow-up bronchoscopy revealed no signs of anastomotic dehiscence or stricture. On a follow-up PET CT, there were no signs of local recurrence or metastatic spread. The patient received no further adjuvant therapy. Now, 6.5 years after surgery, the patient shows no signs of local recurrence.

## 9. Discussion

Carinal resections remain one of the most challenging procedures in thoracic surgery for several reasons. The resection and reconstruction of the airway are surgically complex and are furthermore limited to a certain length, which may affect the completeness of resection. Besides surgical obstacles, ventilation to the contralateral lung must be maintained. There are several options, such as crossfield ventilation and high-frequency jet ventilation, that are considered the gold standard but are inconvenient during the construction of the anastomosis. Moreover, the repeated insertion of the tube into the contralateral bronchus may increase the risk of airway damage. This is why ECMO is an ideal alternative from the surgeon’s perspective [36]. Indications for carinal resections are rare, which is one of the reasons why they should be centralized to high-volume centres [48]. However, as surgeons gain experience in bronchial sleeve resections via open or minimally invasive approaches, carinal resections could be performed in smaller centres with relative safety. Patient selection is crucial, and thorough preoperative planning consisting of CT and PET CT imaging and bronchoscopy and lung and cardiac function testing must be carried out. The aim of these diagnostic procedures is to determine resectability from a surgical but also an oncological point of view, as well as assessing whether the patient has enough functional reserves to be considered operable. In the case of NSCLC, mediastinal staging should be carried out via EBUS or videomediastinoscopy to rule out N2 disease [13]. These patients have poor prognosis and are generally contraindicated for carinal resections. All cases should be discussed at a multidisciplinary tumour board meeting to decide on the best course of action and to offer neoadjuvant or adjuvant treatment if necessary [49]. These procedures have high morbidity rates, which is why patients should be closely monitored for several days after complex airway surgery to avoid minor and major complications or, if they appear, quickly manage them. Airway clearance with bronchoscopy and respiratory physiotherapy is essential. Carinal resections are usually performed via median sternotomy or posterolateral thoracotomy, but in carefully selected patients, minimally invasive techniques such as VATS or RATS have been reported to have great success and low morbidity and mortality [41].

## 10. Conclusions

Complex airway surgery including carinal resections, with or without lung resections, is still burdened with high perioperative mortality and morbidity, although there is a decreasing trend in recent studies thanks to improvements in the fields of surgery, anesthesiology, and intensive care. Most studies are single-institutional and retrospective, spanning several years or decades. It would be difficult to design a randomized controlled trial due to low incidence, ethical concerns, and procedural variations. All of the studies summarized in this article conclude that carinal resections are feasible, with acceptable mortality and morbidity, in carefully selected patients. NSCLC patients with N2 disease are generally contraindicated for this procedure. There are exceptions, such as in single-station N2 disease as part of a multimodality treatment plan discussed at a multidisciplinary tumour meeting. Adenoid cystic carcinoma 5- and 10-year survival rates are promising even in the case of positive margins if adjuvant radiotherapy is feasible. More studies are needed on the role of minimally invasive surgery in these complex procedures. Close cooperation with an experienced anesthesiologist trained in several airway management alternatives is essential. ECMO seems to be an effective option that helps the surgeon in several ways, surpassing crossfield and HFJV in both open and minimally invasive approaches. However larger series and prospective studies are not available, and there is no consensus on absolute indications for ECMO utilization in carinal resections, which is why this option should be carefully considered with regard to potential complications.

## Figures and Tables

**Table 1:** Studies with carinal resections predominantly for NSCLC R0—complete resection with negative margins; RT—radiotherapy; HFJV—high-frequency jet ventilation; N/A—not available; CHT—chemotherapy; CHT/R—chemoradiotherapy; ECMO—extracorporeal membrane oxygenation.

	*n*	Type of Resection	Neoadjuvant	Adjuvant	R0	Ventilation	Incision	Mortality	Morbidity	5-Year OS
Deslauriers	16	Right carinal pneumonectomies	1 RT	0	16	Long single-lumen tube + apneic HFJV	Thoracotomy	25% (5)	62.5% (10)	N/A
Jensik	34	Right carinal pneumonectomies	28 RT	N/A	34	Long single-lumen tube + apneic HFJV	Thoracotomy	29% (10)	41.1% (14)	15%
Dartevelle	55	53 right carinal pneumonectomies 2 left carinal pneumonectomies	5 RT	25 RT 5 CHT	55	HFJV	Thoracotomy	10.9% (6)	12.7% (7)	23%
Mitchell	135	52 carinal resections 44 right carinal pneumonectomies 13 left carinal pneumonectomies 14 carinal sleeve lobectomies 11 carinal resections after prior pneumonectomy	10 RT 4 CHT	N/A	N/A	Crossfield ventilation	Thoracotomy Clamshell	12.7% (17)	39% (52)	N/A
Porhanov	231	162 carinal pneumonectomies 28 carinal resections after prior pneumonectomy 25 carinal resections 15 carinal sleeve lobectomies	39 RT 2 CHT	21 RT 5 CHT	N/A	Crossfield ventilation	Thoracotomy Sternotomy	16% (37)	35.6% (82)	24.7%
Regnard	65	58 right carinal pneumonectomies 2 left carinal pneumonectomies 5 carinal resections	11 CHT	25 RT 4 CHT 4 CHT/R	61	HFJV, crossfield ventilation	Thoracotomy Sternotomy	7.7% (5)	50.7% (33)	26.5%
Macchiarini	50	24 right carinal pneumonectomies 10 left carinal pneumonectomies 5 carinal resections 11 carinal sleeve lobectomies	18 CHT/R	18 CHT	49	Long single-lumen tube Apneic hyperoxygenation technique Crossfield ventilation	Thoracotomy Sternotomy	4% (2)	37% (18)	51%
Roviaro	53	52 right carinal pneumonectomies 1 left carinal pneumonectomy	29 RT, CHT	N/A	N/A	Crossfield ventilation	Thoracotomy	7.5% (4)	11.3% (6)	33.4%
Subotich	42	39 right carinal pneumonectomies 3 left carinal pneumonectomies	5 CHT/R	6 CHT 12 RT	N/A	Crossfield ventilation	Thoracotomy	16.6% (7)	35.7% (15)	35%
Rea	49	48 right carinal pneumonectomies 1 left carinal pneumonectomy	19 RT CHT/R	19 RT CHT/R	N/A	Double-lumen tube + HFJV	Thoracotomy	6.1% (3)	28.6% (14)	27.5%
Jiang	41	41 carinal resections	0	40 CHT	N/A	Crossfield ventilation	Thoracotomy	2.4% (1)	73.1% (30)	26.8%
Eichhorn	64	61 right carinal pneumonectomies 3 left carinal pneumonectomies	5 CHT 3 CHT/R	18 CHT 12 RT 2 CHT/R	53	HFJV	Thoracotomy	3% (2)	41% (26)	31%
Shin	30	17 right carinal pneumonectomies 2 left carinal pneumonectomies 2 carinal resections 9 carinal sleeve lobectomies	2	20 11 RT 5 CHT 4 CHT/R	25	Crossfield ventilation	Thoracotomy	0% (0)	36.6% (11)	66.3%
Costantino	45	14 right carinal pneumonectomies 9 left carinal pneumonectomies 21 carinal resections 1 carinal sleeve lobectomy	8 CHT 6 RT	N/A	20	ECMO Crossfield ventilation	Thoracotomy Sternotomy	6.8% (3)	58% (26)	58%
Herrmann	17	12 right carinal pneumonectomies 5 carinal sleeve lobectomies	N/A	N/A	N/A	Crossfield ventilation	Thoracotomy	17.6% (3)	64.7% (11)	N/A
Sezen	64	51 right carinal pneumonectomies 13 carinal sleeve lobectomies	9 CHT	N/A	56	Crossfield ventilation	Thoracotomy	10.9% (7)	48.8% (31)	42.2%

**Table 2:** Studies with carinal resections for ACC R0, tumour-free resection margins; R1, resection margins containing microscopic tumour; N/A, not available.

First Author, Year	Patients (*n*)	Surgery (*n*)	Complex Surgery	R0 (%)	R1 (%)	Adjuvant Radiotherapy (%)	Overall Survival 5 y–10 y		Disease-Free Survival 5 y–10 y		Local Recurrence (%)	Metastatic Recurrence (%)
Maziak, 1996 [20]	38	32	19 (59.4%)	50	50	78	79	51	N/A	N/A	21	45
Clough, 2006 [21]	13	6	N/A	N/A	N/A	N/A	38.5	N/A	N/A	N/A	N/A	N/A
Zhengjaiang, 2008 [22]	35	28	2 (7.1%)	50	50	60.7	40.2	N/A	N/A	N/A	23	23
Honings, 2010 [23]	108	108	50 (46.3)	9	88	82	78	65	67	53	N/A	N/A
Lee, 2011 [24]	30	17	7 (41.2%)	54	46	59	84	70	N/A	N/A	12	65
Yang, 2016 [25]	109	109	20 (18.4%)	16	84	79	89	43	62	20	12	32
Hogerle, 2019 [26]	38	20	N/A	45	50	65	95	81	69	53	17	47
Wang, 2019 [27]	191	163	N/A	44	44	56	85	63	N/A	N/A	N/A	N/A
Sharma, 2019 [10]	12	6	3 (50.0%)	33	67	100	N/A	N/A	60	33	N/A	N/A
Ning, 2019 [28]	42	42	20 (47.6%)	79	21	N/A	N/A	N/A	N/A	N/A	12	7
Yang, 2020 [29]	132	132	NR	40	60	72	N/A	N/A	N/A	N/A	N/A	N/A
Estephan, 2022 [12]	59	59	38 (64.4%)	29	64	63	82	60	65	47	7	24

## Data Availability

The original contributions presented in this study are included in the article. Further inquiries can be directed to the corresponding authors. No new data were created in this study, and the analyzed data are properly cited in the article.

## Abbreviations

The following abbreviations are used in this manuscript:

ECMO	Extracorporeal Membrane Oxygenation
ACC	Adenoid Cystic Carcinoma
NSCLC	Non-Small Cell Lung Cancer
CT	Computed Tomography
PET CT	Positron Emission Tomography–Computed Tomography
EBUS	Endobronchial Ultrasound
ARDS	Acute Respiratory Distress Syndrome
CPB	Cardiopulmonary Bypass
DLCO	Diffusing Capacity of the Lung for Carbon Monoxide
VATS	Video-Assisted Thoracic Surgery
RATS	Robotic-Assisted Thoracic Surgery
HFJV	High-Frequency Jet Ventilation
V-V	Veno-Venous
V-A	Veno-Arterial
COPD	Chronic Obstructive Pulmonary Disease
VC	Vital Capacity
FEV1	Forced Expiratory Volume in 1 s
ICU	Intensive Care Unit
